# Microbial Ecosystem Therapeutic-2 Intervention in People With Major Depressive Disorder and Generalized Anxiety Disorder: Phase 1, Open-Label Study

**DOI:** 10.2196/32234

**Published:** 2022-01-21

**Authors:** Arthi Chinna Meyyappan, Evan Forth, Roumen Milev

**Affiliations:** 1 Department of Psychiatry Queen's University Kingston, ON Canada; 2 Centre for Neuroscience Studies Queen's University Kingston, ON Canada; 3 Providence Care Hospital Kingston, ON Canada

**Keywords:** gut-brain axis, microbiome, Microbial Ecosystem Therapeutic, depression, anxiety

## Abstract

**Background:**

Recent studies have investigated the potential of treatments that modify the gut microbiome, such as fecal microbiota transplantation and probiotics, in individuals with psychiatric illnesses.

**Objective:**

The aim of this study was to investigate the safety, tolerability, and efficacy of a novel gut microbiome therapeutic, Microbial Ecosystem Therapuetic-2 (MET-2), in people with depression and anxiety.

**Methods:**

In this phase 1, open-label trial, 12 adults diagnosed with major depressive disorder, generalized anxiety disorder, or both were recruited. Over 8 weeks, participants consumed three capsules per day, orally, of an encapsulated microbial therapeutic (MET-2), which contained 40 strains of bacteria that were purified and lab-grown from the stool of a single healthy donor. Participants were assessed biweekly using clinical scales and questionnaires in order to evaluate the safety, efficacy, and tolerability of the therapeutic.

**Results:**

The therapeutic was found to be generally safe and tolerable, with limited adverse events and side effects and no serious adverse events. Of the 12 individuals included in this study, 9 (75%) responded to treatment (50% improvement in Montgomery-Asberg Depression Rating Scale [MADRS] scores, 7-item Generalized Anxiety Disorder scale [GAD-7] scores, or both, from baseline to the week-8 visit). Over the course of 10 weeks, MET-2 significantly decreased mean MADRS and GAD-7 scores (MADRS: *F*_2.731, 30.05_=8.784, *P*<.001; GAD-7: *F*_2.778, 30.55_= 9.638, *P*<.001). Multiple comparisons with Bonferroni adjustments showed a significant reduction in MADRS scores from baseline (mean 19.00, SD 4.843) to week 6 (mean 11.25, SD 8.001; *P*=.009), week 8 (mean 8.667, SD 8.732; *P*=.002), and week 10 (mean 8.250, SD 9.304; *P*=.006). Multiple comparisons showed a significant reduction in GAD-7 scores from baseline (mean 13.58, SD 4.010) to week 4 (mean 9.167, SD 5.096; *P*=.03), week 6 (mean 7.667, SD 4.539; *P*=.004), week 8 (mean 7.333, SD 6.583; *P*=.03), and week 10 (mean 7.500, SD 6.448; *P*=.03).

**Conclusions:**

The findings from this study are the first to provide evidence for the role of microbial ecosystem therapy in treating depression and anxiety. However, a double-blind, randomized controlled trial with a larger sample size is needed for more conclusive results.

**Trial Registration:**

ClinicalTrials.gov NCT04052451; https://www.clinicaltrials.gov/ct2/show/NCT04052451

**International Registered Report Identifier (IRRID):**

RR2-10.2196/17223

## Introduction

Major depressive disorder (MDD) is highly prevalent, affecting over 264 million people of all ages globally [1], and associated with high societal and personal burden. MDD is characterized by persistent depressed mood, loss of interest or pleasure and symptoms that cause clinically significant distress or impairment, or both [2]. MDD is often comorbid with other mental and physical illnesses, such as generalized anxiety disorder (GAD) [3]. GAD is characterized by excessive anxiety and worry about life circumstances, such as work, school, and relationships, among others [4], and has a lifetime prevalence of 5% [3,5]. The psychological symptoms of these illnesses are often accompanied by physical symptoms, such as abdominal issues, pain, and poor sleep quality [6,7]. Though there are a variety of gold standard and novel treatment methods that target symptoms of depression and anxiety [8], the heterogeneity of these disorders has led to difficulty in research in the field of mood and anxiety disorders [9].

Recent research has been exploring the connections between mood and anxiety disorders and the gut microbiome. As such, the gut-brain axis (GBA), which consists of bidirectional signaling between the gastrointestinal (GI) tract and the brain [10], has become a novel target for treatment of mood and anxiety symptoms. Studies suggest that this improvement of depressive and anxiety symptoms and severity may be related to the recolonization of the GI tract with healthy bacteria [11].

The purpose of this study was to evaluate the safety, efficacy, and tolerability of a GBA treatment method known as Microbial Ecosystem Therapeutic-2 (MET-2). MET-2 comes in an encapsulated form and is composed of 40 purified strains of lyophilized bacteria from a healthy 25-year-old donor; this was chosen for its favorable safety profile. MET-2 was developed in response to the growing body of literature supporting the ameliorative effects of fecal microbiota transplantation (FMT) on symptoms of depression. FMT is a procedure used to recolonize a patient’s gut microbiota through the transplantation of feces from a donor to the recipient. A recent systematic review details the literature to date surrounding the safety, efficacy, and mechanisms of action for FMT [11]. Though FMT has been found to be effective in many cases, it is still an arduous, expensive, and invasive procedure. MET-2 provides an exciting alternative to FMT, with the possibility of conferring the same ameliorative properties with an easier and more tolerable mode of delivery. The objectives of this study were to assess the safety, tolerability, and efficacy of MET-2.

## Methods

### Study Design and Ethics Approval

This study was a 10-week, open-label, phase 1 clinical trial conducted out of Providence Care Hospital in Kingston, Ontario, Canada. This study was approved by the Health Science Regulatory Ethics Board of Queen’s University, Ontario, Canada, and all methods were performed in accordance with the Declaration of Helsinki. The protocol has been previously published [11,12]. This study was registered with ClinicalTrials.gov on August 9, 2019 (NCT04052451).

### Participants

Inclusion criteria for participants were as follows: (1) aged 18 to 65 years; (2) a diagnosis of MDD, GAD, or both, using the Mini International Neuropsychiatric Interview (MINI); (3) and no current use of any antidepressant medications. Mood, anxiety, sleep, GI symptoms, and severity of illness were assessed at screening using the Montgomery-Asberg Depression Rating Scale (MADRS) and the 7-item Generalized Anxiety Disorder scale (GAD-7). A minimum score of 15 on the MADRS or 8 on the GAD-7 were also required for inclusion in the study. For the full set of inclusion, exclusion, and discontinuation criteria, as well as the detailed study design, see the previously published protocol [12]. Participants were recruited from the local community using posters and online advertisements. Signed written informed consent was obtained from all participants in this study for the collection of all forms of data.

### Intervention

The investigational product in this study was MET-2: capsules composed of 40 purified strains of lyophilized bacteria from a healthy 25-year-old donor. MET-2 was developed by NuBiyota in Guelph, Ontario, Canada. During the 8 weeks of treatment, all participants consumed three MET-2 capsules per day orally; each 0.5-g MET-2 capsule contains 3.2 × 10^5^ to 3.2 × 10^11^ colony-forming units. This was known as the maintenance dose. Additionally, a loading dose of 5 g of MET-2 was taken for 2 days immediately following baseline and week-2 visits for all participants and following the week-4 visit for nonresponders (ie, those lacking a reduction in MADRS or GAD-7 scores by 50% by this time point) [12]. Mood, anxiety, GI symptoms, and sleep quality were assessed at all biweekly treatment visits. At the week-10 follow-up, only mood and anxiety were assessed.

### Outcome Measures

The clinical measures included the following: (1) the GAD-7 [13] to assess anxiety symptoms and severity, with scores ranging from 0 (no anxiety) to 21 (severe anxiety); (2) the MADRS [14] to assess depressive symptoms and severity, with scores ranging from 0 (no depression) to 60 (severe depression); (3) the Snaith-Hamilton Pleasure Scale (SHAPS) [15] to assess anhedonia, with scores ranging from 0 (no anhedonia) to 14 (severe anhedonia); (4) the 16-item Quick Inventory of Depressive Symptomatology–Self-Report (QIDS-SR16) [16] to assess depressive symptoms, with scores ranging from 0 (no depression) to 27 (severe depression); (5) the Gastrointestinal Symptom Rating Scale (GSRS) [17] to assess GI symptoms, composed of five subscales—reflux, diarrhea, constipation, abdominal pain, and indigestion syndrome—with scores ranging from 1 (no discomfort) to 7 (severe discomfort); (6) the Pittsburgh Sleep Quality Index (PSQI) [18] to assess subjective sleep quality, with scores ranging from 0 (poor sleep quality) to 21 (good sleep quality); and (7) the Clinical Global Impressions-Severity scale (CGI-S) [19] to assess illness severity, with scores ranging from 0 (no illness) to 7 (severe illness).

Participants used a personal mood and symptom log to track any new symptoms that they have been experiencing since the beginning of treatment [20], assess the tolerability of treatment, and keep track of their mood and sleep. Adverse events were assessed and recorded at all visits; they were categorized by frequency, severity, and causality. Only adverse events rated as a grade 2 or above were included in the analysis. Investigational product safety was assessed via recorded symptoms on the Toronto Side Effects Scale (TSES) [21-23], which is a 31-symptom scale with each symptom having a frequency and severity score ranging from 1 (never, no trouble) to 5 (every day, extreme trouble), respectively; the personal logs; and adverse events [12].

### Statistical Analysis

Prism (version 8; GraphPad Software) was used to analyze all data from clinical measures obtained throughout the study and to create plots. A repeated-measures analysis of variance (ANOVA) was used to analyze changes in clinical measures from baseline to week 10. Paired *t* tests were used to compare the means of clinical measures at each time point to baseline. If a participant returned after a first course of treatment and later withdrew, their final clinical scores were projected to week 10.

### Availability of Data and Materials

The data sets generated or analyzed during this study are not publicly available as of yet, but they are available from the corresponding author on reasonable request.

## Results

### Study Population

The final study cohort consisted of 12 participants, 8 (67%) of whom were female, with a total mean age of 28.8 (SD 12.8) years. The participants were recruited from May 16 to November 7, 2019. The trial profile can be found in Figure S1 in Multimedia Appendix 1. A total of 21 participants were originally screened for the study; 7 (33%) were ineligible due to a lack of MDD or GAD diagnosis or presence of mania, as per the MINI. Out of the remaining 14 participants, 2 (14%) withdrew prior to the week-2 visit for personal reasons and were not included in the analysis. The study population was diverse, with ages ranging from 19 to 59 years and representation from four different ethnicities. Further demographic information can be found in Table 1. Out of 12 participants, 10 (83%) were diagnosed with both MDD and GAD; 6 of these 10 (60%) were currently experiencing a major depressive episode, with the remainder having experienced at least one major depressive episode in the past. The 2 remaining participants out of 12 (17%) had sole diagnoses of MDD or GAD, respectively. All participants were combined into one group for analysis due to the high comorbidity of the two psychiatric illnesses. Mean baseline MADRS and GAD-7 scores were 19.0 (SD 4.8) and 13.6 (SD 4.0), respectively.

**Table 1:** Participant demographics.

Characteristic			Participants (N=12), n (%)	
**Gender**				
	Male	4 (33)		
	Female	8 (67)		
**Diagnosis**				
	Major depressive disorder (MDD) only	1 (8)		
	Generalized anxiety disorder (GAD) only	1 (8)		
	MDD and GAD	10 (83)		
**Ethnicity**				
	Caucasian	8 (67)		
	Chinese	2 (17)		
	Latin American	1 (8)		
	South Asian	1 (8)		
**Education level**				
	High school graduate or some college	3 (25)		
	College or university degree	9 (75)		
**Employment status**				
	Student	7 (58)		
	Working	2 (17)		
	On leave or disability	3 (25)		

### Efficacy Measures

The principal efficacy measures used were the MADRS, GAD-7, and CGI-S. A one-way repeated-measures ANOVA showed significant reductions in mean MADRS scores between visits (*F*_2.731, 30.05_=8.784, *P*<.001). Multiple comparisons with Bonferroni adjustments showed a significant reduction in MADRS scores from baseline (mean 19.00, SD 4.843) to week 6 (mean 11.25, SD 8.001; *P*=.009), week 8 (mean 8.667, SD 8.732; *P*=.002), and week 10 (mean 8.250, SD 9.304; *P*=.006). There was a slight reduction in MADRS scores from baseline to week 2 (mean 14.67, SD 6.946; *P*=.08) and week 4 (mean 13.42, SD 9.443; *P*=.20); however, neither was significant. Additionally, 8 out of 12 (67%) participants were responders and improved by at least 50% in MADRS scores by week 8, of whom 7 (88%) remained responders by week 10. Of those who remained responders (n=7), 1 (14%) still worsened in mood symptoms, but not below 50% from baseline.

Similarly, results from the one-way repeated-measures ANOVA showed a significant reduction in mean GAD-7 scores (*F*_2.778, 30.55_=9.638, *P*<.001). Multiple comparisons showed a significant reduction in GAD-7 scores from baseline (mean 13.58, SD 4.010) to week 4 (mean 9.167, SD 5.096; *P*=.03), week 6 (mean 7.667, SD 4.539; *P*=.004), week 8 (mean 7.333, SD 6.583; *P*=.03), and week 10 (mean 7.500, SD 6.448; *P*=.03). There was a slight reduction in GAD-7 scores from baseline to week 2 (mean 10.92, SD 4.542), which was not significant (*P*=.51). Additionally, 7 out of 12 (58%) participants were responders, and 6 (50%) improved by at least 50% in GAD-7 scores by week 8, of whom 5 (83%) remained responders by week 10. Of those who remained responders (n=5), 1 (20%) still worsened in anxiety symptoms, but not below 50% from baseline. A significant reduction in mean CGI-S scores was seen (*F*_2.833, 31.17_=8.709, *P*<.001) from baseline (mean 3.667, SD 0.7785) to week 6 (mean 2.667, SD 0.9847; *P*=.006) and week 8 (mean 2.333, SD 1.073; *P*=.001). Although a slight reduction was seen in CGI-S scores from baseline to week 2 (mean 3.250, SD 0.7538; *P*=.26) and week 4 (mean 3.250, SD 0.9653; *P*=.56), neither was significant.

Additional efficacy measures included the QIDS-SR16, SHAPS, and PSQI, three self-report measures evaluating depressive symptomology, anhedonia, and subjective sleep quality, respectively. QIDS-SR16 scores were found to be significantly reduced (*F*_2.402, 26.42_=7.111, *P*=.002) from baseline (mean 12.42, SD 3.147) to week 6 (mean 7.333, SD 5.836; *P*=.007) and week 8 (mean 6.750, SD 6.166; *P*=.005). Reductions from baseline to week 2 (mean 11.17, SD 4.877; *P*=.25) and week 4 (mean 8.667, SD 6.315; *P*=.10) were not significant.

No significant reduction in mean SHAPS scores was found (*F*_2.166, 23.82_=0.9579, *P*=.40) from baseline (mean 3.917, SD 3.988) to week 2 (mean 3.333, SD 3.651), week 4 (mean 2.917, SD 4.641), week 6 (mean 3.417, SD 4.358), or week 8 (mean 2.583, SD 3.919).

Results from tests of between-subject contrasts in a one-way repeated-measures ANOVA showed a significant reduction in mean PSQI scores (*F*_2.547, 28.02_= 3.100, *P*=.05). Multiple comparisons between PSQI scores at baseline (mean 9.333, SD 2.839) and week 2 (mean 8.500, SD 3.000), week 4 (mean 7.083, SD 3 .988), and week 6 (mean 7.917, SD 3.450) were not significant, but there was significance between PSQI scores at baseline (mean 9.333, SD 2.839) and week 8 (mean 7.000, SD 3.838). Graphs showing the change in efficacy outcome measures over the course of the study can be found in Figures S2-S7 in Multimedia Appendix 1.

### Safety and Tolerability Measures

A total of 11 adverse events and zero serious adverse events were reported by participants during the course of the study. The majority of these adverse events were declared unrelated to the investigational product by the participants’ family physicians, the principal investigator, or both. The most common adverse event was a stomachache, but this was reported multiple times by the same participant. Only one reported adverse event was considered to have a possible relationship to the study product: an instance of a stomachache rated level 2 with moderate pain. The full list of reported adverse events can be found in Table 2. As MET-2 is a therapeutic targeting the gut, its effect on GI symptoms was measured with the GSRS at five time points during the course of the study. One-way repeated-measures ANOVA showed no significant change in mean GSRS scores (*F*_1.998, 21.98_=1.451, *P*=.26) from baseline (mean 1.871, SD 0.9754 ) to week 2 (mean 1.663, SD 0.5375), week 4 (mean 1.514, SD 0.5338), week 6 (mean 1.539, SD 0.6699), or week 8 (mean 1.514, SD 0.6273). A graph depicting the change in GSRS scores over the course of the study can be found in Figure S8 in Multimedia Appendix 1.

Side effect frequency and severity was measured using the TSES. A total of 31 side effects with an intensity greater than 14 were reported. These side effects can be found in Table 3.

**Table 2:** Adverse events reported by participants.

Adverse event	Incidence, n
Stomachache	3
Rash or black dots	1
Eye pain	1
Bloating	2
Diarrhea	1
Lower abdomen cramps	1
Itchy throat	1
Vomiting	1
Anxiety attack	1
Panicky feeling or jitters	1
Heart palpitations	2
Nightmares	1
Abdominal pain	1

**Table 3:** Side effects with an intensity greater than 14, as measured by the Toronto Side Effects Scale (TSES).

Side effect^a^	Incidence, n
Nervousness	5
Agitation	3
Tremor	2
Dyspepsia	1
Nausea	1
Diarrhea	1
Weakness or fatigue	3
Drowsiness	2
Increased sleep	1
Decreased sleep	1
Flushing	3
Headache	1
Dry mouth	1
Anorgasmia	1
Decreased libido	1
Delayed ejaculation	1
Impotence	1
Bloating	1
Heart palpitations	1

^a^Each symptom measured by the TSES had a frequency and severity score ranging from 1 (never, no trouble) to 5 (every day, extreme trouble), respectively.

## Discussion

### Principal Findings

In this study, MET-2, the novel gut microbiota–targeting treatment for symptoms of MDD and GAD, was found to be safe, generally tolerable, and efficacious. These findings are in line with what was expected, according to the literature to date regarding FMT, and suggest that gut microbiome manipulation can result in the alleviation of symptoms of a variety of psychiatric illnesses, including MDD and GAD. Preclinical research has found both the conferment of psychiatric symptoms through FMT from animals displaying behaviors related to psychiatric illnesses to antibiotic-treated animals [24-27] and the amelioration of psychiatric symptoms after FMT from healthy animals to those displaying psychiatric symptoms [28,29]. Clinical research has found that the transfer of microbiota from a healthy donor to an ill recipient often results in the alleviation of psychiatric symptoms [11]. As MET-2 is a collection of bacteria cultured from the gut of a healthy human donor, it was suspected, and subsequently supported by this study, that treatment with MET-2 would result in symptom improvement similar to that seen in clinical FMT studies.

When comparing studies, MET-2 was found to be as safe as FMT, since both the FMT studies and this study reported relatively few adverse events that were deemed to be related to the treatment. When comparing the tolerability and burden to the patient, it is expected that MET-2 would be less of a burden, as FMT can be a rather arduous process, but further research would be needed to determine which treatment is more feasible.

### Limitations

This study addresses some of the limitations in the literature around GBA treatments, such as fecal transplants, including safety, stigma, and labor costs. It also evaluates the use of a GBA treatment without other treatment interference, such as antidepressants or structured psychotherapy. The main limitation of this study, as with the reviewed clinical studies, was the small sample size and open-label design. The lack of large-scale, double-blind randomized controlled trials makes it difficult to determine efficacy and safety. The small sample size, in addition to the missing data, prevents large-scale analyses between parameters and may be the reason for the limited significance in the data, given the trends that were seen. Additionally, the lack of a placebo arm, in conjunction with the limited power in the sample size, suggests that the results may be merely due to the placebo effect or chance. That said, typically, the placebo effect is around 30% to 40% in psychiatric indications, and our study response was 75%, suggesting that it was unlikely to be a placebo effect. It also seems that the effects of MET-2 may begin to wear off after stopping treatment; however, given our study was only 10 weeks long and the follow-up period was only 2 weeks long, we cannot be certain at what point and rate the effects diminish. The follow-up period in future studies will need to be longer to determine if there is a need for maintenance therapy.

Further, given the nature of the study, participants were asked to come in every 2 weeks for an in-person visit, where, inevitably, they had an opportunity to open up about their mood and related symptoms, which could be therapeutic in and of itself. This could have contributed to the quick improvement in mood and anxiety symptoms.

Finally, though we looked to see whether participants may have had a diagnosis for an eating disorder or alcohol dependence, we did not ask how often these individuals were drinking or what their eating habits were. These components may have had an effect on the response to, or the transiency, of treatment.

### Conclusions

In summary, our study has found MET-2 to be efficacious, as it significantly improved mood and anxiety symptoms in as early as 4 and 2 weeks of treatment, respectively. We found that 9 out of 12 (75%) participants had improved by at least 50% in their MADRS or GAD-7 scores from the start to the end of treatment. This improvement was seen in conjunction with limited side effects and a lack of serious adverse events.

With high individual variability in symptomatology and prognosis, high concentrations of comorbidity with other disorders, and genetic and environmental influences, progress in research in the treatment of psychiatric disorders has been challenging. Given the adaptable nature of the gut microbiome, it may be a good representation of the individual’s history and could explain differences in risk of illness, disease course, and response to treatment.

With the complicated heterogeneity of psychiatric disorders, finding one treatment that works for all patients is not achievable, especially given the range of factors that influence the disorder and treatment response. Our study has shown MET-2 to considerably improve mood and anxiety symptoms, with limited side effects. While the research in this field is far from complete, the potential of targeting the GBA using GBA treatments, such as FMT and MET-2, to alleviate symptoms of psychiatric illness is promising. That said, further large-scale research in exclusively psychiatric indications is needed to strengthen the evidence that gut repopulation treatments, specifically MET-2, can be effective treatment methods.

## Appendix

Multimedia Appendix 1 Trial profile and graphs of outcome measures.

## Abbreviations

ANOVA: analysis of variance
CGI-S: Clinical Global Impressions-Severity scale
FMT: fecal microbiota transplantation
GAD: generalized anxiety disorder
GAD-7: 7-item Generalized Anxiety Disorder scale
GBA: gut-brain axis
GI: gastrointestinal
GSRS: Gastrointestinal Symptom Rating Scale
MADRS: Montgomery-Asberg Depression Rating Scale
MDD: major depressive disorder
MET-2: Microbial Ecosystem Therapeutic-2
MINI: Mini International Neuropsychiatric Interview
PSQI: Pittsburgh Sleep Quality Index
QIDS-SR16: 16-item Quick Inventory of Depressive Symptomatology–Self-Report
SHAPS: Snaith-Hamilton Pleasure Scale
TSES: Toronto Side Effects Scale
