# Supplementary material for: Microbial Ecosystem Therapeutic-2 Intervention in People With Major Depressive Disorder and Generalized Anxiety Disorder: Phase 1, Open-Label Study
Source: Interact J Med Res. 2022 Jan 21;11(1):e32234. doi: 10.2196/32234 (PMC8817211; doi:10.2196/32234)
Supplement: Multimedia Appendix 1 [file ijmr_v11i1e32234_app1.docx]

**Multimedia Appendix 1. Trial profile and graphs of outcome measures**

**Figure S1.** Trial profile: A total of 21 participants were screened for the study, 7 were ineligible due to lack of MDD/GAD diagnosis or presence of mania, as per MINI. Study population consists of anyone that attended more than one post-baseline (first treatment) visit. Two participants withdrew prior to this visit for personal reasons and were not included in analysis.


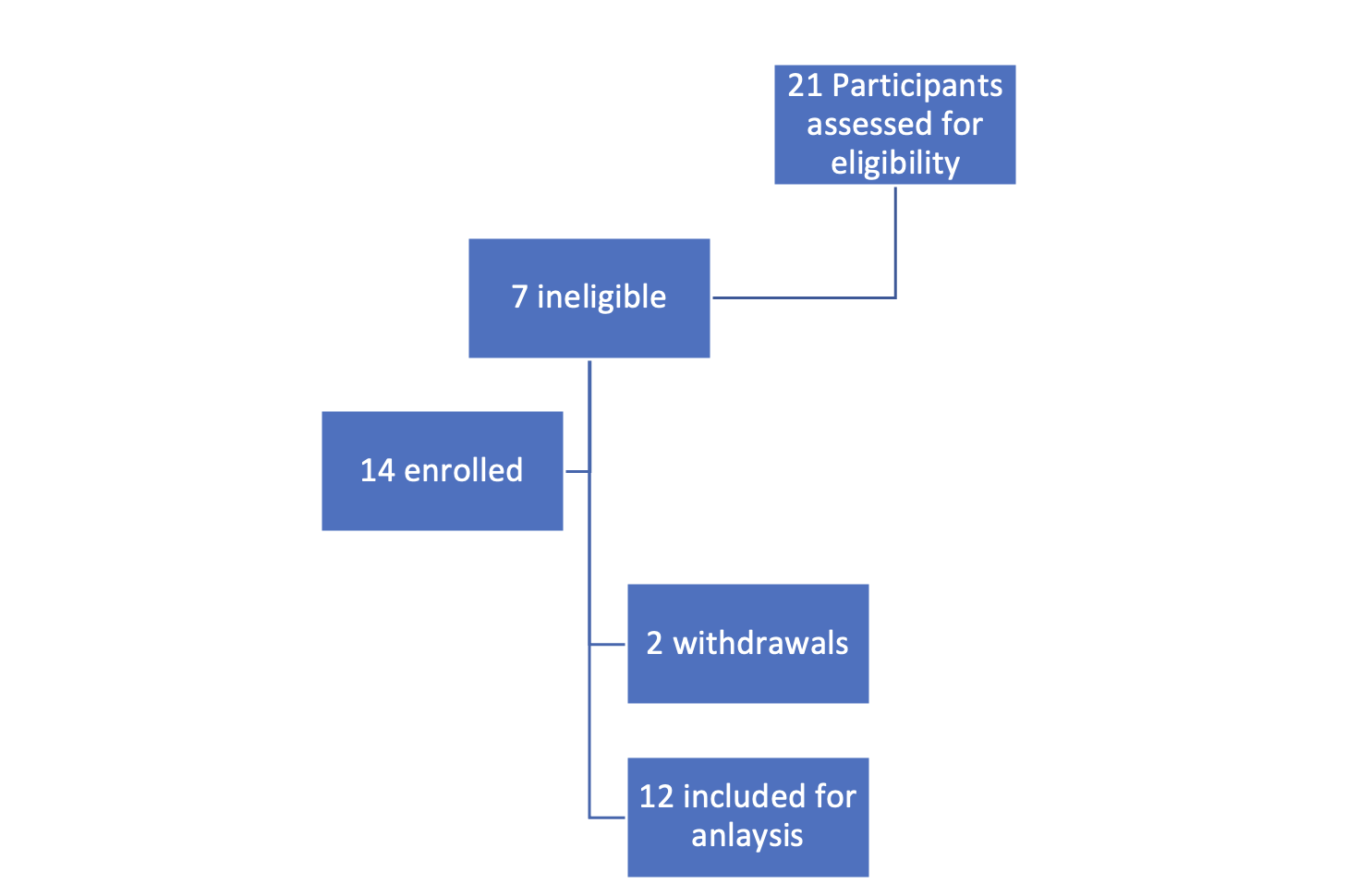


**Figure S2.** Change in mean severity of illness (CGI-S) score

**Figure S3.** Change in mean sleep (PSQI) score

**Figure S4.** Change in mean mood (MADRS) score

**Figure S5.** Change in mean anxiety (GAD-7) score

**Figure S6.** Change in mean self-rated mood (QIDS-SR16) score

**Figure S7.** Change in mean anhedonia (SHAPS) score

**Figure S8.** Mean gastrointestinal symptom (GSRS) scores over time
